# Investigating the added effects of guidance in digital psychological self-care for alcohol problems (ALVA)—protocol for a randomized factorial optimization trial

**DOI:** 10.1186/s13063-024-07981-6

**Published:** 2024-02-21

**Authors:** Christopher Sundström, Ekaterina Ivanova, Philip Lindner, Magnus Johansson, Martin Kraepelien

**Affiliations:** 1 Centre for Psychiatry Research, Department of Clinical Neuroscience, Karolinska Institutet & Stockholm Health Care Services, Stockholm, Sweden; 2 Department of Psychology, Uppsala University, Uppsala, Sweden

**Keywords:** Alcohol, Digital, Self-guided, Intervention, Optimization, Factorial experiment

## Abstract

**Background:**

The continual development and implementation of effective digital interventions is one important strategy that may serve to bridge the well-known treatment gap related to problematic alcohol use. Research suggests that clinician guidance, provided in different ways during the digital intervention (i.e., written weekly messages, phone calls etc.), can boost intervention engagement and effects. *Digital psychological self-care* (DPSC) is a new delivery format wherein an unguided digital intervention is provided within the framework of a structured care process that includes initial clinical assessment and follow-up interviews. In a recent feasibility study, a DPSC intervention for problematic alcohol use, ALVA, provided without any extra guidance, was found safe and credible and to have promising within-group effects on alcohol consumption. The aim of the current study is to gather information on the effects and efficiency of different forms of guidance added to ALVA, in order to optimize the intervention.

**Methods:**

This protocol describes a randomized factorial trial where the effects of two different ways of providing guidance (mid-treatment interview, weekly written messages, respectively) in DPSC for problematic alcohol use are investigated. Optimization criteria will be applied to the results regarding how effective the intervention is at reducing alcohol consumption measured by the number of standard drinks per week together with the clinician time spent on guidance.

**Discussion:**

This study will investigate the added benefit of different forms of guidance to DPSC for problematic alcohol use. These added effects will be compared to the added cost of guidance, according to pre-defined optimization criteria.

**Trial registration:**

Clinicaltrials.gov: NCT05649982. Registered on 06 December 2022. Prospectively registered.

## Administrative information

Note: the numbers in curly brackets in this protocol refer to SPIRIT checklist item numbers. The order of the items has been modified to group similar items (see http://www.equator-network.org/reporting-guidelines/spirit-2013-statement-defining-standard-protocol-items-for-clinical-trials/).

**Table:** 

Title {1}	Investigating the added effects of guidance in digital psychological self-care for alcohol problems (ALVA) – protocol for a randomized factorial optimization trial
Trial registration {2a and 2b}.	Clinicaltrials.gov: NCT05649982. Registered on 06 December 2022. Prospectively registered.
Protocol version {3}	Version 1. June 30 2023
Funding {4}	FORTE (grant number 2020-01160), Fredrik O Ingrid Thurings stiftelse (grant number 2019-00470), Swedish Ministry of Health and Social Affairs (grant number S2018/03855/FS)
Author details {5a}	^1^Centre for Psychiatry Research, Department of Clinical Neuroscience, Karolinska Institutet & Stockholm Health Care Services, Stockholm, Sweden ^2^Department of Psychology, Uppsala University, Uppsala, Sweden
Name and contact information for the trial sponsor {5b}	Centre for Psychiatry Research, Department of Clinical Neuroscience, Karolinska Institutet & Stockholm Health Care Services, Norra Stationsgatan 69, 113 64 Stockholm, Sweden
Role of sponsor {5c}	The sponsor played no part in study design; and will play no part in the collection, management, analysis, and interpretation of data; writing of the report; and the decision to submit the report for publication.

## Introduction

### Background and rationale {6a}

Although alcohol problems are widespread and constitute a major burden on global health [1], only one in six seek help [2]. Digital interventions for alcohol problems are effective in reducing alcohol consumption and could serve to bridge this treatment gap [3]. In the largest meta-analysis conducted to date, guidance from a therapist or coach was found to boost effects of digital interventions for alcohol problems [4]. Guidance usually means that some sort of human contact is provided to the user. However, the way that the contact is provided differs in studies. The most common way guidance is provided in digital interventions is through weekly written messages exchanged on the digital platform on which the intervention is provided [5]. Other forms of guidance include synchronous chat messages [6], phone calls [7], and face-to-face sessions [8]. Although the meta-analysis from 2018 mentioned above found guidance to boost effects, added effects of guidance have been absent in some recent trials [5, 9–11]. Furthermore, a recent study showed that a guided digital alcohol intervention was non-inferior to traditional face-to-face cognitive behavioral therapy, when provided in a routine clinical setting [12].

### Digital psychological self-care—a new delivery format

Digital psychological self-care (DPSC) is a new proposed delivery format wherein an essentially self-guided digital intervention is provided within the framework of a structured care process that includes an initial clinical assessment interview with a clinician and one or several follow-up interviews [13]. The efficacy of well-designed self-guided digital interventions in depression and anxiety has in some studies been shown to be comparable to digital interventions with therapist guidance [14, 15]. Crucial for the improvement, however, seems to be a clear clinical process that employs clinical assessments and follow-up interviews, some form of clinical monitoring during the treatment, automated written support, and a material of high quality designed for the user to easily be able to guide themselves through the intervention [14]. The concept of DPSC is presented in a 2023 paper by Kraepelien and colleagues [16].

We recently conducted a small feasibility study (*n* = 36) on a basic version of DPSC for problematic alcohol use named ALVA [13]. We found not only that the intervention was safe and credible but also that it rendered a high degree of use. In terms of effects, participants reduced their alcohol consumption from 22.6 standard drinks preceding week before the digital intervention to 10.6 standard drinks after the intervention (Hedge’s g intra-group = 0.85) and 12.7 standard drinks at a 3-month follow-up (Hedge’s g intra group = 0.70). The clinicians in the study spent less than 1 h on average per participant, time which was mainly spent on the initial clinical assessment interview and the post-intervention interview [13]. There was also a follow-up interview in the feasibility study, but this was not an essential part of the DPSC concept.

For the study described in this protocol, we propose that the basic version of DPSC for problematic alcohol use should include the following:

1. An initial clinical assessment interview with a clinician who informs about DPSC and refers the client to appropriate health care services should DPSC be deemed not appropriate
2. A digital intervention based on evidence-based theory with high quality texts, illustrations, exercises, patient narratives, automated written support, and interfaces
3. Provision of immediate technical support for the client in the event of practical problems when interacting with the digital intervention
4. A clear routine for monitoring the clients’ weekly self-assessments of alcohol consumption and symptom levels, including indications of suicidal ideation
5. Possibility for clients to flag themselves in the event of a crisis or problem, which will lead to immediate contact with a clinician. When in doubt, DPSC for the client should be reconsidered
6. A post-intervention interview where the client is asked about experiences and where the need for additional help is investigated

We also propose that 1 h of clinician-time per participant would signal high scalability and that it would be easy to communicate to health care providers that the intervention would be relatively easy to implement.

### Objectives {7}

The objective is to gather information on the effects and efficiency of different forms of guidance added to the basic version of digital psychological self-care for problematic alcohol use (ALVA) as tested in the earlier study, in order to optimize [17] the intervention for a future randomized controlled trial (RCT).

### Research questions

1. What are the main and interaction effects of the two guidance forms investigated (i.e., mid-treatment phone call, weekly written messages) on alcohol consumption, immediately after the intervention and up to 12 months after the intervention?
2. Which combination of guidance forms is most efficient (i.e., renders the greatest effects on alcohol consumption per additional cost in the form of incremental resource consumption measured by clinician time), immediately after the intervention and up to 12 months after the intervention?
3. Which (combination of) guidance form(s) gives the best effects on alcohol consumption given a set limit of 1 h of clinician time per participant, immediately after the intervention and up to 12 months after the intervention?

### Trial design {8}

The purpose of an optimization trial and the corresponding phase within the framework of the Multiphase Optimization Strategy is to provide the information needed to make decisions about which components to select for the optimized intervention, which will later be tested in a classic RCT [17]. The framework is exploratory and follows a decision-priority perspective, in contrast to the conclusion-priority perspective of a RCT. Although scientific conclusions are of interest, the top priority in a decision-priority perspective is to make practical decisions about which components and component-levels to select [17]. The current protocol describes a randomized factorial optimization trial [18] where the added effects of two different forms of guidance (a mid-treatment phone call interview and weekly written guidance) to the basic version of ALVA will be investigated. Optimization criteria [17] will include both additional effects on alcohol consumption and associated consumption of clinician time. The two two-level factors will result in four experimental conditions with an allocation of 1:1:1:1.

## Methods: participants, interventions, and outcomes

### Study setting {9}

The study will be conducted at the research clinic at the Centre for Psychiatry Research, Stockholm Health Care Services, and Karolinska Institutet, Sweden, in cooperation with the Addiction eClinic at the Stockholm Centre for Dependency Disorders, Sweden. Participants will be recruited nationally in Sweden via advertisements on the patient community and support website Alkoholhjalpen.se as well as through search engines and social media. The participants log in to the intervention website at egenvard.webcbt.se.

### Eligibility criteria {10}

Eligibility is both based on the screening (certain cut-off scores) and determined in the interview by the interviewer in a clinical manner. To be included in the trial, participants must (a) be ≥ 18 years, (b) have regular Internet access, and (c) score 8 points or more for men and 6 points or more for women on the Alcohol Use Disorders Identification Test (AUDIT) [19]. Potential participants will be excluded from the trial if they display (a) insufficient knowledge of Swedish, (b) reading and/or writing difficulties to the extent that it makes it difficult to participate in the intervention, (c) other ongoing psychological treatment with a content similar to that in the current study, (d) high suicide risk based on the clinical assessment interview, or (e) other urgent need for more intensive psychiatric or addiction care, based on the clinical assessment interview. Potential participants excluded will be referred to, or informed about, more appropriate care options in their regional area and condition-related area.

### Who will take informed consent? {26a}

All participants will receive detailed written information about the study and provide informed consent digitally on the study platform BASS when applying for the study. Online informed consent to participate will be obtained from all participants.

### Additional consent provisions for collection and use of participant data and biological specimens {26b}

There will be no additional consent for ancillary studies.

### Interventions

#### Explanation for the choice of comparators {6b}

The experimental factors are weekly written guidance and/or a mid-treatment interview, added to the basic version of ALVA. As stated above in the “Background and rationale {6a}” section, the existent literature is inconsistent on the added effect of including weekly written guidance in digital alcohol interventions, with one meta-analysis claiming that guidance boosts effects on alcohol consumption [4], while recent trials have failed to demonstrate such added effects of guidance [5, 9–11]. Written guidance consumes around 15 min clinician time per participant and week in the study of the earlier mentioned comprehensive intervention [5]. An added extra interview by phone could also boost engagement and effects, especially for participants in risk of treatment failure [20]. The basic version of ALVA (including two phone interviews) includes the participant setting new goals for alcohol consumption mid-treatment; guidance in the form of an extra phone interview may help with this potentially difficult task.

#### Intervention description {11a}

The intervention, ALVA, consists of techniques from cognitive behavioral therapy (CBT) and relapse prevention (see Table 1) given during 8 weeks. The intervention is centered around the alcohol diary. The alcohol diary guides participants to set goals for their alcohol consumption for 4 weeks at a time. Every morning, a text-message reminds the participant to log in and register the previous day’s consumption in the alcohol diary. The alcohol-related goals will be defined in three ways:

**Table 1:** The content of ALVA

Component	Description
Daily alcohol diary	The digital tool for goal setting and daily registration of standardized drinks. Goal fulfillment is calculated automatically and provided back to the user with along with a visual presentation
Weekly module 1	Psychoeducation regarding alcohol and alcohol problems, reasons to change, and goal setting. Introduction of patient narratives and instructions on how to use the alcohol diary. Summary of important strategies more comprehensively introduced in weeks 2–4
Weekly module 2	Identify risk situations and strategies for handling them
Weekly module 3	Refusal skills and strategies for drinking moderately
Weekly module 4	Strategies for scheduling alternative activities, maintaining progress, and relapse prevention
Weekly module 5	Evaluate progress and set a new goal for the next 4 weeks
Weekly module 6	Encouragement to continue using the alcohol diary and strategies. New patient narrative texts
Weekly module 7	Encouragement to continue using the alcohol diary and strategies. New patient narrative texts
Weekly module 8	Encouragement to continue using the alcohol diary and strategies. New patient narrative texts
Maintenance plan	Construct a long-term maintenance plan and advice on relapse prevention

- Number of standardized drinks per day
- Number of standardized drinks per week
- Number of abstinent days per week

Additional exercises include dealing with cravings, refusal skills, and finding alternative activities. After the end of the intervention, a telephone interview will be conducted where the participant will receive feedback regarding their work with the exercises and the alcohol diary. The feedback is intended to act as a motivation to continue to work with the exercises after the intervention’s 8 weeks. For a more detailed description of the basic version of the intervention, see the previous study [13]. The experimental additions to the basic version of ALVA are detailed below.

The experimental factors are as follows:

- Factor 1. Weekly guidance: Written guidance similar to more comprehensive guided interventions [5] aiming at a maximum of 15 min a week per participant
- Factor 2. Mid-treatment interview: Focus on goal fulfillment and setting of a new 4-week goal, motivation and encouragement to continue adhere to the program, aiming at around 20 to 30 min per participant

The two experimental factors result in four different combinations (Table 2).

**Table 2:** Factorial trial design with target sample per group

		Factor 2: Mid-treatment phone call		
		Yes	No	Total
Factor 1: Weekly written guidance	Yes	75	75	150
	No	75	75	150
Total		150	150	300

#### Criteria for discontinuing or modifying allocated interventions {11b}

Since the experimental factors are related to support, all extra support given to a participant could be said to slightly alter the allocated interventions. The suicide ideation item from self-rated MADRS-S [21] will alert the clinician monitoring the intervention if rated 4 out of 6 or over. The clinician will contact participants with suicidal ideation, or who describes severe withdrawal symptoms, by phone. This time will be measured as clinician time. There is also a possibility for all participants to alert the clinician within the system to be contacted by the study administration for technical support. All time spent on extra support for a participant will be registered. The intervention can be discontinued based on participant request, if it appears to lead to serious adverse events, or if the participant requires medical care incompatible with the intervention provided in the trial.

#### Strategies to improve adherence to interventions {11c}

Automatic text-message reminders to use the alcohol diary will be administered daily. There will also be separate reminders for self-rated assessments. If a participant is a week late with an assessment, the study administration may make a short call to the participant as a reminder. All time spent on calls will be registered.

#### Relevant concomitant care permitted or prohibited during the trial {11d}

Receiving other care during the intervention is not grounds for exclusion. The use of other, parallel care will be assessed after the intervention.

#### Provisions for post-trial care {30}

Participants with a continued need for care will be referred to different levels of care provided by the Stockholm Centre for Dependency Disorders, including digital interventions open to residents of all Swedish regions. Participants may also be encouraged to seek care within the municipality or the region to which they belong.

#### Outcomes {12}

Measurements will be made at registration (screening), before (week 0), during (week 4), and after the 8-week intervention (primary end-point, week 8), as well as at a 3-month follow-up (FU3), 6-month follow-up (FU6), and lastly at a 12-month follow-up (FU12). Measurements will take place in the form of self-assessment surveys via the secure digital treatment platform and supplemented with interview data from telephone interviews at week 0, week 8, and FU6. If there are sufficient resources available, a telephone interview may also be done at FU12.

### Primary outcome

The main outcome will be changes from baseline in standardized drinks (corresponding to 12 g of ethanol) per week, self-rated (number of drinks the previous week) at the post intervention time point. A decreased number of drinks per week is associated with improved quality of life and fewer alcohol-related consequences. Optimization criteria will be applied to the primary outcome regarding how effective the intervention is at reducing alcohol consumption at week 8 and how this is related with the clinician time spent on guidance (see the “Research questions” section).

### Secondary outcomes

Diagnostic criteria Alcohol Use Disorder [22]

Credibility/Expectancy Questionnaire [23]

Alcohol Use Disorders Identification Test [19]

Brunnsviken Brief Quality of Life Scale [24]

Penn Alcohol Craving Scale [25]

Patient Health Questionnaire 9 [26]

Generalized Anxiety Disorder 7 [27]

Readiness To Change Questionnaire—treatment version [28]

Self-rated questions about any adverse effects of the intervention

**Fig. 1:**
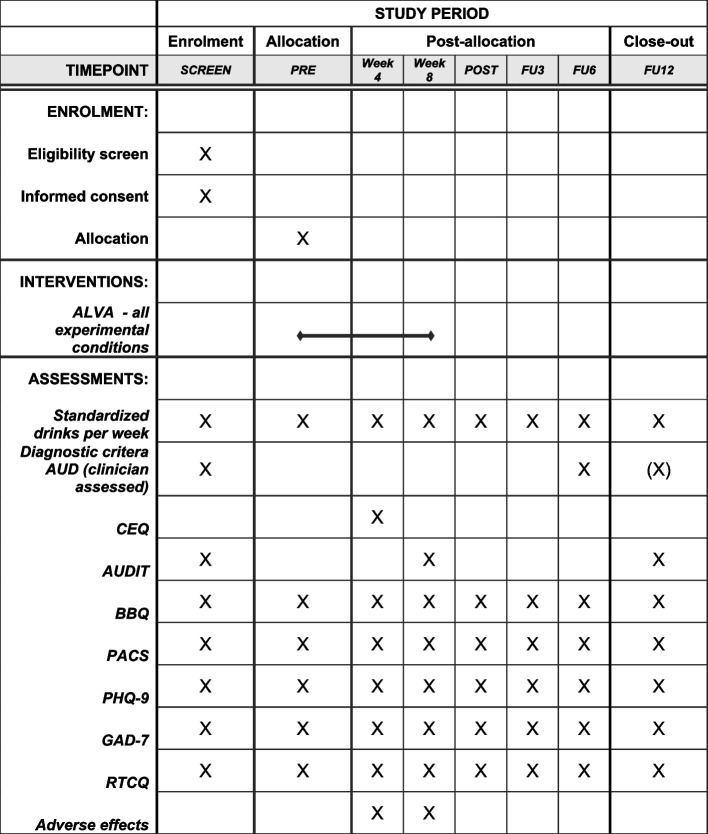
Schedule of enrollment, intervention, and assessments. ALVA, name of intervention; AUD, alcohol use disorder; CEQ, Credibility/Expectancy Questionnaire; AUDIT, Alcohol Use Disorders Identification Test; BBQ, Brunnsviken Brief Quality of Life Scale; PACS, Penn Alcohol Craving Scale; PHQ-9, Patient Health Questionnaire 9; GAD-7, Generalized Anxiety Disorder 7; RTCQ, Readiness To Change Questionnaire—treatment version

See Fig. 1 for time points for all outcomes.

#### Participant timeline {13}

See Fig. 1 for a detailed time schedule of enrollment, intervention, and assessments. If there are sufficient resources available for a telephone interview at FU12, we will also perform a diagnostic interview of alcohol use disorder at the FU12 time point.

#### Sample size {14}

Statistical power was calculated using the R-package MOST for factorial optimization studies [29] to be able to detect a main between-group effect corresponding to Cohen’s *d* = 0.35. This calculation showed that 259 participants is sufficient. A small to moderate between-group effect size of 0.35 has been used in previous studies of support functions such as guidance in digital interventions [11, 30]. In a meta-analysis of digital interventions for problem drinking, guided interventions rendered 6.8 standard drink greater reductions than unguided interventions, which also speaks for a small to moderate effect of guidance [4]. With a margin for data loss, we aim to recruit a total of up to 300 participants. See Table 2 for target sample per group.

#### Recruitment {15}

As mentioned above under the “Study setting {9}” section, participants will be recruited nationally in Sweden via advertisements on the patient community and support website Alkoholhjalpen.se as well as on digital search services or social media. Advertisements achieved a satisfying rate of participant enrollment in the previous smaller study of the intervention [13].

### Assignment of interventions: allocation

#### Sequence generation {16a}

After inclusion, participants will be allocated randomly (1:1) to factor 1 (mid-treatment phone call) as well as (1:1) to factor 2 (weekly written guidance), resulting in four even arms (1:1:1:1). Randomization will be based on a true random number generator (http://www.random.org). There is no stratification of the randomization procedure.

#### Concealment mechanism {16b}

The allocation sequence will be generated with the true random number generator by a person not otherwise involved with the study.

#### Implementation {16c}

The study administrators (MK, CS, and other clinical psychologists or psychology students with basic training in psychotherapy) will enroll participants. The allocation sequence will then be generated by a person blinded to the participants and who is not otherwise involved with the study. The study administrators will then assign the participants to the intervention groups in the digital platform according to the allocation sequence.

### Assignment of interventions: blinding

#### Who will be blinded {17a}

There will be no blinding of participants, care providers, or assessors, but to minimize placebo/nocebo effects, the information to participants is that all participants will receive the active intervention with only some differences in added guidance. The assigned mode of added guidance will be explained to the participant after randomization by the study administration and within the intervention. The main outcomes are self-rated by the participants. The data analysist will be blinded.

#### Procedure for unblinding if needed {17b}

N/A. No unblinding will be needed because there will be no blinding.

### Data collection and management

#### Plans for assessment and collection of outcomes {18a}

Self-rated assessments will be made at registration (screening), before (week 0), during (week 4), after the 8-week intervention (week 8), at a 3-month follow-up (FU3), 6-month follow-up (FU6), and lastly at a 12-month follow-up (FU12). Telephone interviews will be made by the study administrators at week 0, week 8, and FU6. If there are sufficient resources available, a telephone interview may also be done at FU12.

#### Plans to promote participant retention and complete follow-up {18b}

If data is missing for the primary outcome of standardized drinks per week at week 8, FU6 and possibly at FU12, the number of standardized drinks the preceding week will be assessed in the telephone interview and used to replace the missing data point. Telephone-administered self-report data has been shown to be able to reduce the impact of missing data [31].

#### Data management {19}

All self-rated data will be entered by the participant and stored at a secure server at Karolinska Institutet. Interview data will be entered by the study administrator interviewing the participant into the same technical platform as the self-rated data and stored on the secure server at Karolinska Institutet. This study will use the BASS platform from the eHealth Core Facility at Karolinska Institutet for data management. Double data entry is not possible on the BASS platform; rather, all data is time-stamped. Range check will be conducted. No other data quality processes will be used.

#### Confidentiality {27}

All data will be will be collected and stored according to Good Clinical Practice, on a secure digital platform provided by the eHealth Core Facility at Karolinska Institutet. The data will be made available to other researchers upon reasonable request.

#### Plans for collection, laboratory evaluation, and storage of biological specimens for genetic or molecular analysis in this trial/future use {33}

N/A. There will be no collection of biological specimens.

### Statistical methods

#### Statistical methods for primary and secondary outcomes {20a}

Main and interaction effects will be estimated based on all available measurement points using mixed models with the appropriate family function. Incremental cost-effectiveness ratios will be calculated where the main effect in spent clinician time of a factor is divided by the main effect in alcohol consumption. The uncertainty in data for incremental ratios will be handled using nonparametric bootstrapping. An optimization criterion with the best possible effect on alcohol consumption given a maximum of 1 h of clinician time per participant will also be explored in order to select an optimal combination of guidance.

#### Interim analyses {21b}

There will possibly be interim analyses for clinical psychology student master theses, but no decision to terminate the trial will be based on the results from any student thesis.

### Methods for additional analyses (e.g., subgroup analyses) {20b}

We will explore who gained from the intervention under different levels of added guidance with regard to gender, age, and severity of alcohol problems.

#### Methods in analysis to handle protocol non-adherence and any statistical methods to handle missing data {20c}

Analysis will be intention-to-treat, i.e., without consideration of non-adherence and with estimation of missing data. We intend to conduct random effects modeling or similar statistical analyses which uses all available data to estimate missing data, without the need for imputation.

#### Plans to give access to the full protocol, participant-level data, and statistical code {31c}

Non-identifiable participant-level data may be available on reasonable request to the principal investigator, author MK.

### Oversight and monitoring

#### Composition of the coordinating center and trial steering committee {5d}

The trial team coordinating the study, including the principal investigator, will meet weekly during phases of the trial with active participants. The larger project managing group, including clinical and statistical expertise, will meet every 3–6 months.

#### Composition of the data monitoring committee, its role and reporting structure {21a}

There will be no data monitoring committee, as there is a low risk of harm and mainly self-rated data. Data integrity is secured by it being submitted to the secure technical platform at Karolinska Institutet as described under the “Data management {19}” section.

#### Adverse event reporting and harms {22}

Any adverse effects of the intervention will be measured at weeks 4 and 8 (during and immediately after the intervention) using self-rated questions. Adverse events in behavioral interventions usually entail stress or anxiety related to initiating a behavior change. No regulatory bodies will be contacted.

#### Frequency and plans for auditing trial conduct {23}

There will be no independent auditing of trial conduct.

#### Plans for communicating important protocol amendments to relevant parties (e.g., trial participants, ethical committees) {25}

Any significant changes to the protocol require the trial coordinators to seek permission from the Swedish Ethical Review Authority.

#### Dissemination plans {31a}

Dissemination of trial results will include conference presentations and journal publications. All journal publications will be made publicly available with open access.

## Discussion

The published feasibility study [13] showed the potential of digital psychological self-care being safe, credible, and preliminary moderately effective in reducing alcohol consumption. This promising result was despite the base intervention being provided without any additional guidance other than around 1 h of clinician time spent on phone interviews. The present study could help finding the optimal level and mode of guidance to be given with the digital self-care intervention. This may be an important step towards implementing scalable, yet effective, interventions for individuals with problematic alcohol use within existing health care services.

### Trial status

Version 1, June 30, 2023. Recruitment started on January 11, 2023, and is expected to end in Q4 2024.

## Abbreviations

DPSC: Digital psychological self-care
ALVA: A digital psychological self-care intervention for problematic alcohol use
RCT: Randomized controlled trial
CBT: Cognitive behavioral therapy
AUD: Alcohol use disorder
CEQ: Credibility/Expectancy Questionnaire
AUDIT: Alcohol Use Disorders Identification Test
BBQ: Brunnsviken Brief Quality of Life Scale
PACS: Penn Alcohol Craving Scale
PHQ-9: Patient Health Questionnaire 9
GAD-7: Generalized Anxiety Disorder 7
RTCQ: Readiness To Change Questionnaire—treatment version
BASS: Technical platform at the eHealth Core Facility at Karolinska Institutet
FU3: Three-month follow-up
FU6: Six-month follow-up
FU12: Twelve-month follow-up
